# Study on the Mechanical Properties of Carbon Fabric/Polyetherketoneketone Composites Under Different Environmental Conditions

**DOI:** 10.3390/polym17091142

**Published:** 2025-04-22

**Authors:** Xiangyu Xu, Baoyan Zhang, Fenghui Shi, Kai Liu, Gongqiu Peng, Junpeng Gao

**Affiliations:** 1 AVIC Manufacturing Technology Institute Composite Technology Center, Beijing 101300, China; 2 School of Aerospace Engineering, Beijing Institute of Technology, Beijing 100081, China

**Keywords:** PEKK, thermoplastic composite, bending performance, shear performance, hygrothermal aging, CFRP, carbon fabric

## Abstract

Carbon fabric reinforced polyetherketoneketone (CFF/PEKK) composites have garnered significant attention from researchers due to their superior properties and have been successfully applied in various engineering fields. Environmental conditions are known to directly influence the mechanical properties and service life of composites; however, limited literature exists on the mechanical behavior of CFF/PEKK composites under different environmental conditions. This study elucidates the correlation between the bending and shear behaviors of CFF/PEKK composites and environmental factors, thereby offering robust data support for engineering applications. In this work, CFF/PEKK composite laminates with a fiber volume fraction of 55 vol% were fabricated and subjected to saturated moisture absorption treatments at 70 °C. The moisture absorption characteristics of the material were investigated. The bending and shear properties of CFF/PEKK composites were characterized under three environmental conditions: −55 °C dry state (CTD), room temperature dry state (RTD), and 70 °C wet state (ETW). Failure modes and mechanisms of composite specimens were also analyzed. The equilibrium moisture absorption rate of CFF/PEKK composites is approximately 0.27%. Hygrothermal aging resulted in noticeable fiber pull-out in mechanical specimens, indicating damage to the interfacial performance of the composites. Furthermore, no cracks or delamination were observed. Results indicate that in the CTD condition, the bending strength and shear strength of CFF/PEKK composites are higher compared to those in the RTD condition, while the modulus remains relatively unaffected. In the ETW condition, both bending and shear properties exhibit a significant decline, with the most pronounced reduction observed in interlaminar shear strength. No significant differences in failure modes were noted across different environmental conditions.

## 1. Introduction

Carbon fiber reinforced polymer composites (CFRPs) are extensively utilized in aerospace, automotive, wind power, shipbuilding, and other industries, owing to its superior mechanical properties, excellent corrosion resistance, and outstanding fatigue resistance [1,2,3]. Currently, in the aviation industry, thermosetting composites are utilized across various components of aircrafts and have achieved significant weight reduction benefits [4,5]. With successive launches of programs such as European Thermoplastic Affordable Primary Aircraft Structure (TAPAS), TAPAS 2, Clean Sky, and Clean Sky 2, civil aviation structures have put forward higher requirements for the economy and environmental protection of aviation materials [6,7]. However, thermosetting composites are increasingly unable to meet the economic and environmental requirements of the aviation industry. Compared with commonly used thermosetting composites, thermoplastic composites offer distinct advantages such as recyclability, enhanced design flexibility, lower cost, and shorter molding cycles, thereby attracting significant attention from researchers [8,9].

A wide variety of resin matrices are available for thermoplastic composites, including polyetheretherketone (PEEK), polyphenylene sulfide (PPS), polyetherimide (PEI), polyetherketoneketone (PEKK), and polyethersulfone (PES) [10,11,12]. The molecular structure of PEKK, characterized by the orderly arrangement of benzene rings, ether bonds, and ketone groups, confers exceptional molecular stability. Additionally, its broader processing window makes it particularly suitable for automated forming processes such as Automated Fiber Placement (AFP) [13,14]. These attributes have established PEKK as one of the most widely utilized high-performance thermoplastic resin matrices. For example, the fuselage of the Airbus A350 incorporates composite materials made from PPS and PEEK, with Tenax TPCL laminates utilizing their TPWF thermoplastic woven fabric prepregs. PEKK exhibits distinct advantages in terms of environmental resistance [15]. Specifically, the glass transition temperature (Tg) of PEKK ranges from 156 to 165 °C, which is significantly higher than that of PEEK (143 °C) [16]. This characteristic enables PEKK to maintain superior rigidity and dimensional stability at elevated temperatures. Furthermore, the brittle transition temperature of PEEK is as low as −60 °C, which contributes to its excellent low-temperature toughness.

Fiber-reinforced resin matrix composites (FRPs) inevitably encounter complex environmental factors, including temperature, humidity, radiation, wind and sand erosion, and chemical corrosion during service. These factors lead to the degradation of the material’s mechanical properties. Since both temperature and humidity can significantly influence the mechanical properties, hygrothermal aging is one of the primary failure modes for FRP [17,18]. Therefore, in addition to conventional research on mechanical properties, the investigation of composite performance under various environmental conditions is also a current research hotspot. Li et al. [19] investigated the pin-loaded tensile behavior and failure behaviors of carbon-fiber-reinforced polyetherketoneketone (CF/PEKK) composites at various temperatures. Their findings revealed the exceptional bearing load resistance of CF/PEKK composites, supporting their suitability for high-performance structural applications. Yildirim et al. [20] reported that high temperature and cyclic hygrothermal conditions significantly reduce both mode-I and mode-II fracture toughness compared to room temperature, whereas low temperature conditions enhance mode-II toughness despite decreasing mode-I toughness. Pedoto et al. [21] reported a behavior similarity between PEKK polymer and CF/PEKK composites, particularly in terms of non-linearity, temperature and time dependency, and analog response to creep and recovery solicitation. According to Yun et al. [22], the fracture toughness of thermoplastic laminates were reduced by 67.5%, 72.4%, and 85.1% at temperatures of 40 °C, 60 °C, and 80 °C, respectively, compared to the room temperature. Dong et al. [23] summarized six life prediction models for the four thermoplastic polymers-based composites under different environmental exposures. Micelli et al. [24] noted that each type of FRP has distinct components and manufacturing processes; consequently, conclusions drawn for one material may not be applicable to others. This variability is a primary reason for the differences in testing methods and results observed in numerous aging studies on FRP.

Due to the significant dispersion of test data across various studies, it is challenging to achieve consistent results, and there remains a lack of a comprehensive test database on the durability of composite materials. In our prior study, we examined the effects of wet heat aging on the tensile and compressive properties of CFF/PEEK composites; however, changes under low-temperature conditions were not addressed [25]. Despite their chemical structural similarity, PEEK and PEKK demonstrate significant differences in processability and mechanical performance when serving as resin matrices for CFRP. Currently, CFF/PEKK composites have found extensive application in the aerospace domain. Nevertheless, substantial gaps remain in the mechanical performance data of these composites under varying environmental conditions, and the lack of such data may directly constrain their practical engineering applications. While researchers have conducted extensive studies on the wet heat aging behavior of composites, systematic comparisons and analyses of the mechanical properties of CFF/PEKK composites under different environmental conditions are still lacking. Thus, this study investigates the bending and shear behaviors of CFF/PEKK composites under diverse environmental conditions, with the objective of further enriching the comprehensiveness of the thermoplastic composite database.

In this study, CFF/PEKK composite laminates with a volume fraction of approximately 55 vol% were fabricated and subjected to saturation moisture absorption treatments at 70 °C. The bending and shear properties of the composites were characterized under dry conditions at −55 °C (CTD), room temperature (RTD), and wet conditions at 70 °C (ETW). The moisture absorption behavior, failure modes, and damage mechanisms of the specimens were analyzed in detail. Through comprehensive data analysis, the moisture absorption characteristics of CFF/PEKK composites and the correlations between their mechanical properties and environmental conditions were elucidated. This research provides reliable mechanical property data for CFF/PEKK composites and elucidates their evolution under different environmental conditions, thereby offering a more accurate reflection of material performance in real service environments. These findings can further expand the application scope of thermoplastic composites.

## 2. Materials and Methods

### 2.1. Materials

Satin carbon fabric and PEEK resin were employed as the raw materials to fabricate CFF/PEEK thermoplastic prepreg. The prepreg was developed by the AVIC Manufacturing Technology Institute Composite Technology Center (Beijing, China). The PEEK resin was sourced from Zhejiang Pfulon Advanced Materials Co., Ltd. (Quzhou, Zhejiang, China). The satin carbon fabric, characterized by a unit area mass of 293 ± 8 g/m², was supplied by Weihai Expand Fiber Co., Ltd. (Weihai, Shandong, China).

### 2.2. Preparation of Specimen

**Figure 1:**
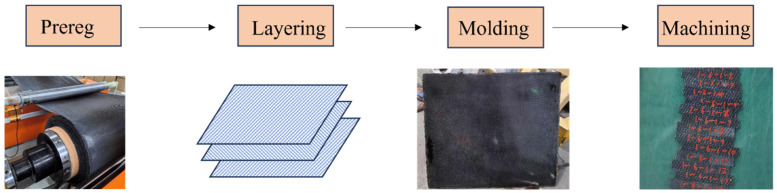
Schematic illustration of the test specimen preparation procedure.

After cutting the prepregs to the appropriate dimensions, manual layup was performed according to the specified configurations detailed in Table 1. The layed-up prepregs were then placed into a mold and hot-pressed using a high-temperature press to fabricate CFF/PEKK composite laminates. The procedure for preparing test specimens is illustrated in Figure 1. Finally, the composite laminates were mechanically processed to the standard dimensions as required by the testing standards to obtain the mechanical performance specimens, with specific dimensions also provided in Table 1. Mechanical property verification was conducted on three batches of prepregs.

### 2.3. Hydrothermal Treatment

To fulfill the requirements for aircraft material selection, the hygrothermal aging temperature for the CFF/PEKK composite is established at 70 °C in compliance with the provisions specified in the aviation industry standard HB 7618 [26]. The water absorption performance of the composite was monitored using the gravimetric method in accordance with ASTM D5229 [27]. The mechanical specimens and moisture-absorbing specimens were immersed together in deionized water. At each aging interval, the moisture-absorbing specimens were removed from the water bath, surface moisture was blotted dry with absorbent paper, and then immediately weighed on an electronic balance with a precision of 0.1 mg. The weight measurements were recorded. Five specimens were tested for each group, and the average value was calculated. The water absorption rate of the composite material at time t can be determined using Equation (1).

(1) $$M_{t}=\frac{W_{t}-W_{0}}{W_{0}}\times 100\%$$

where $W_{t}$ is the mass of the sample at time *t*; and $W_{0}$ is the initial mass of the sample.

### 2.4. Mechanical Characterization

**Figure 2:**
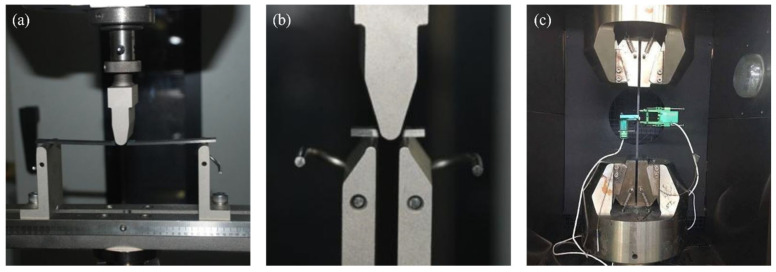
Schematic illustrations of mechanical property tests for composite materials: (**a**) bending test; (**b**) interlaminar shear test; (**c**) in-plane shear test.

The bending performance of the composite materials was evaluated using a Zwick Z2.5/Z010 electronic universal testing machine in accordance with ASTM D7264 [28]. The interlaminar shear strength was tested following ASTM D2344 [29], and the in-plane shear properties were assessed according to ASTM D3518 [30]. For the bending and interfacial shear tests, the loading rate was set to 1 mm/min, while for the in-plane shear test, it was 2 mm/min. The schematic diagram of the mechanical tests for the composite materials is presented in Figure 2. The test environment temperature was controlled using a WGDN-7350 high and low-temperature chamber produced by Lishi (Shanghai) Scientific Instrument Co., Ltd. (Shanghai, China).

To facilitate a direct comparative analysis of the mechanical property test results, it is essential to normalize the original experimental data to the performance values corresponding to the specified fiber volume fraction, as presented in Equation (2). Normalizing the properties that are predominantly influenced by fibers can effectively minimize data dispersion.

(2) $$X_{N}=\frac{t_{T}}{t_{N}}X_{T}$$

where *X_N_* denotes the normalized value of mechanical properties; *t_T_* represents the measured thickness of the specimen; *t_N_* corresponds to the nominal thickness of the specimen; and *X_T_* indicates the experimental value obtained from the mechanical property test.

Each test group comprises six specimens. Following the normalization of their raw data using Formula (2), the mean value $\bar{X}$ (Formula (3)) and standard deviation S (Formula (4)) are subsequently computed for each group.

(3) $$\bar{X}=\frac{\sum_{i=1}^{n}X_{i}}{n}$$

(4) $$S=\sqrt{\left(\sum X^{2}-n{\bar{X}}^{2})/(n-1\right)}$$

where *X* denotes the performance test value for each trial, and *n* represents the total number of specimens.

### 2.5. Morphological Analysis

The microscopic morphologies of the fracture surfaces of the mechanical specimens were examined both before and after hygrothermal aging using a Quanta 450 FEG field emission environmental scanning electron microscope (SEM) manufactured by Thermo Fisher Scientific (Waltham, MA, USA).

## 3. Results

### 3.1. Moisture Kinetics

Since the samples were subjected to edge banding prior to being placed in the aging environment, the diffusion behavior of water molecules within the test samples can be regarded as one-dimensional diffusion without boundary effects. The diffusion behavior of water molecules within the composite can be characterized using the water transport kinetics equation, as presented in Equation (5).

*M_t_*/*M_m_* = *kt^n^* (5)

where *M_t_* is the moisture absorption rate at time *t*, *M_m_* is the saturated moisture absorption rate, *k* is the diffusion constant of water, and *n* is a critical parameter that characterizes the swelling mechanism of the material.

The CFF/PEKK composite specimens were subjected to saturated moisture absorption treatment at 70 °C. The *k* and *n* obtained from fitting the moisture absorption data of CFF/PEKK composite using the logarithmic form of the aforementioned equation are illustrated in Table 2. Analysis of these data reveals that the value of n for moisture absorption curve is approximately 0.5. Therefore, it can be reasonably inferred that the initial water absorption behavior of CFF/PEKK composite during the early stages of aging is consistent with the Fickian diffusion model [31,32].

The solution to Fickian diffusion model can be approximately represented as follows:

(6) $$\frac{M_{t}}{M_{m}}=1-\frac{8}{\pi^{2}}\sum_{n=0}^{n=\infty }\frac{1}{{\left(2n+1\right)}^{2}}exp\left[-\left(\frac{Dt}{h^{2}}\right)\pi^{2}{\left(2n+1\right)}^{2}\right]$$

where *M_t_* and *M_m_* represent the water absorption rate and the saturated water absorption rate of the sample at time *t*, respectively; *n* is the parameter describing the swelling mechanism; and *h* is the thickness of the specimen.

**Figure 3:**
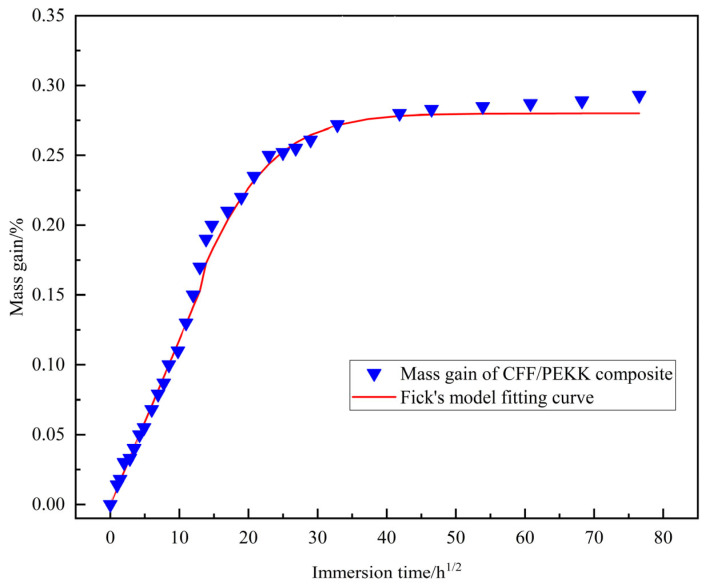
Water absorption curves and Fickian fitting results of CFF/PEKK composite.

Based on the parameters derived from Table 2 of the experiment, the Fickian model fitting the curve for CFF/PEKK composite was generated and compared with the measured moisture absorption data, as illustrated in Figure 3. The equilibrium moisture absorption rate for CFF/PEEK composite is approximately 0.26 wt%. Kitamoto et al. [33] have reported that the moisture absorption of the unidirectional carbon fiber/epoxy composite (T700S/2592, Toray (Osaka, Japan)) at 40 °C increased continuously, reaching 0.9 wt%, and subsequently continued to rise without exhibiting any clear signs of moisture absorption saturation. Wu et al. [34] found that the saturated moisture absorption rate of T300/epoxy composite material at 70 °C is approximately 0.782 wt%, markedly lower than that of glass fiber-reinforced and flax fiber-reinforced epoxy resin composites. Based on these findings, it can be reasonably inferred that under identical conditions, the saturated moisture absorption of CFF/PEKK composites is significantly lower than that of thermosetting CFRP, indicating their superior humidity resistance properties.

The distribution of the fitting curves and data points in Figure 2 indicates that during the initial stage of moisture absorption, the experimental data exhibit excellent agreement with the Fickian model. The curve gradient is relatively steep, indicating a linear relationship between the moisture absorption rate and *t*^1/2^. In the later stages of moisture absorption, the curve deviates from linearity. This phenomenon may be attributed to the influence of the material’s internal microstructure, such as pores, cracks, and interfacial debonding, on moisture absorption in composite materials, which alters the diffusion pathways of water molecules. Furthermore, Fick’s law assumes that water molecules diffuse freely, which may contribute to the discrepancy between the theoretical model and experimental data. Given that carbon fibers consist mainly of carbon elements with a disordered graphite structure, they exhibit minimal water absorption [35]. The moisture absorption of composite materials primarily involves the resin matrix and the interface between fibers and the resin [36]. PEKK resin, as a high-performance engineering plastic, features an aromatic ring structure and high crystallinity. Consequently, under conventional moisture exposure conditions, the likelihood of hydrolysis is minimal, with water molecules predominantly diffusing within the amorphous regions of the resin. Prolonged exposure to hygrothermal aging environments can lead to the formation of hydrogen or chemical bonds between water molecules and the polymer matrix.

### 3.2. Bending Properties

**Figure 4:**
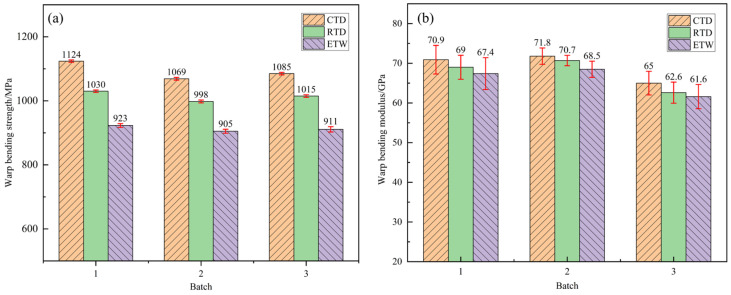
Bending property of the warp direction of CFF/PEKK composite under different environmental conditions: (**a**) warp bending strength; (**b**) warp bending modulus.

**Figure 5:**
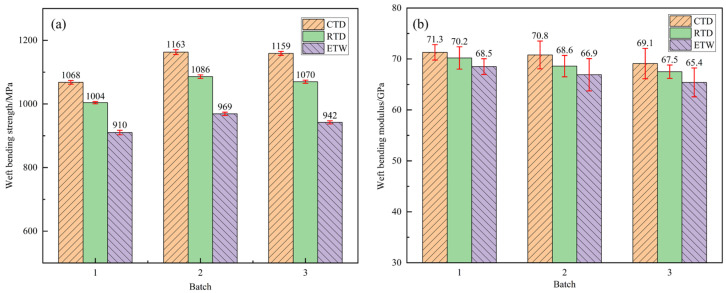
Bending property of the weft direction of CFF/PEKK composite under different environmental conditions: (**a**) weft bending strength; (**b**) weft bending modulus.

The bending properties of CFF/PEKK composites under CTD, RTD, and ETW conditions were evaluated. The warp-direction bending properties are presented in Figure 4, while the weft-direction bending properties are shown in Figure 5. Table 3 and Table 4 provide the retention rates of bending properties under different environmental conditions. As indicated by the data, the bending performance of CFF/PEKK composites improved significantly in the RTD state: the retention rates of bending strength in the warp and weft directions were 107.71% and 107.26%, respectively, compared to the RTD state; the retention rates of bending modulus were 102.71% and 102.38%, respectively. This enhancement can be attributed to the restricted movement of resin molecular chains at low temperatures, which increases matrix rigidity [37]. Additionally, low temperatures promote higher crystallinity in the resin matrix, leading to a denser material structure and improved stiffness and strength. Moreover, carbon fibers typically exhibit increased strength and modulus at low temperatures, potentially enhancing the interfacial bonding between fibers and resin. After wet heat aging, the bending properties in the ETW state showed a significant decline: the retention rates of bending strength in the warp and weft directions were 90.01% and 89.30%, respectively, relative to the RTD state; the retention rates of bending modulus were 97.66% and 97.33%. Water molecules entering the resin matrix act as plasticizers, reducing intermolecular forces within the resin. Furthermore, water molecules may form hydrogen bonds with polar groups such as carbonyl (C=O) and ether (-O-) in the resin matrix, facilitating further water diffusion and degrading matrix properties. Elevated temperatures accelerate water molecule diffusion, exacerbating the degradation of both matrix and interface properties, thereby reducing bending performance. Overall, bending strength is more sensitive to environmental conditions than bending modulus, which exhibits relatively minor variations across the three environmental states.

**Figure 6:**
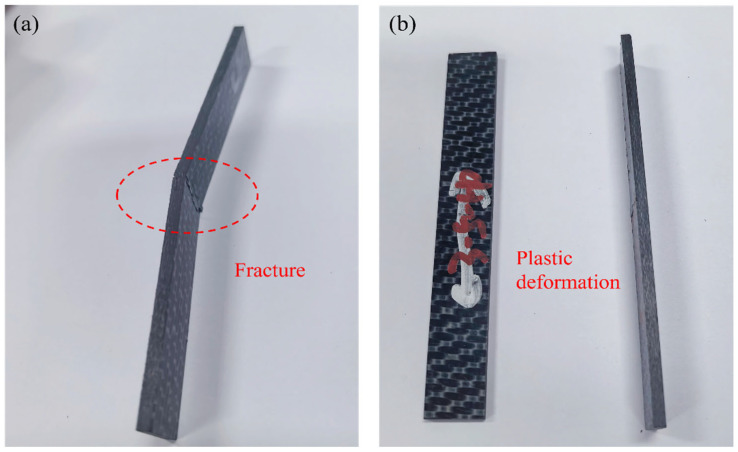
Failed bending specimens of CFF/PEKK composite: (**a**) fracture; (**b**) plastic deformation.

The failure modes in bending tests can manifest in various forms, including matrix cracking, fiber breakage, delamination, interfacial debonding, plastic deformation, or mixed failure [38]. The failure modes of CFF/PEKK composite bending specimens were analyzed, as illustrated in Figure 6. Observations of the failed specimens revealed no significant differences in failure modes across the CTD, RTD, and ETW states. A small number of specimens exhibited mixed failure characterized by fiber fracture and delamination at the mid-span position, as shown in Figure 6a. In contrast, most specimens did not exhibit obvious damage after the bending test but primarily showed plastic deformation, as depicted in Figure 6b. This behavior is attributed to instability in the fibers or laminate structure within the loading zone, while the plasticity of the matrix delayed buckling, resulting in permanent plastic deformation.

### 3.3. In-Plane Shear Properties

**Figure 7:**
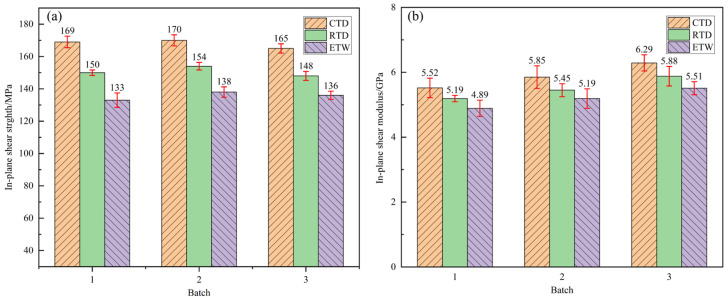
In-plane shear property of CFF/PEKK composites under different environmental conditions: (**a**) in-plane shear strength; (**b**) in-plane shear modulus.

In-plane shear performance is a critical indicator for assessing the interface performance between fibers and resin in CFRP, reflecting the material’s ability to resist shear failure in engineering applications. The in-plane shear properties of CFF/REKK composites under RTD, CTD, and ETW conditions are presented in Figure 7, while Table 5 shows the retention rates of these properties. As indicated by the data, the shear performance of CFF/REKK composites under CTD conditions has significantly improved, with strength reaching approximately 111.52% and modulus reaching approximately 106.89% of that under RTD conditions. At low temperatures, the thermal motion of molecular chains in the resin matrix is suppressed, enhancing matrix rigidity and significantly improving resistance to shear deformation. Additionally, the increased brittleness at low temperatures may temporarily increase crack propagation resistance, restricting lateral crack expansion and forcing shear failure along the primary fiber direction, thereby increasing shear strength. Under ETW conditions, the in-plane shear performance of CFF/REKK composites decreases, with strength retention at approximately 90.06% and modulus retention at approximately 94.39% of RTD conditions. The data reveal that the variation in in-plane shear modulus under different environmental conditions is notably smaller compared to the variation in shear strength. In hygrothermal aging environments, the combined effects of humidity and temperature accelerate water molecule diffusion within the composite, leading to faster degradation of matrix and interface performance [39]. Long-term exposure to such conditions results in cumulative damage, potentially triggering interface fatigue cracking or fiber stress corrosion, ultimately reducing the overall shear performance of the composite material.

**Figure 8:**
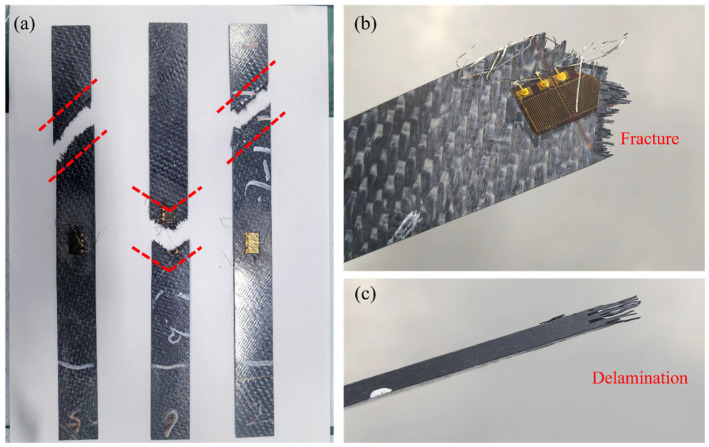
Failed in-plane shear specimens of CFF/PEKK composites: (**a**) overall morphological characteristics; (**b**) fracture; (**c**) delamination.

**Figure 9:**
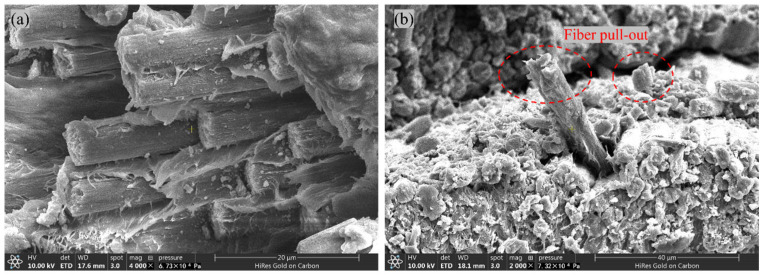
SEM micrographs of the fracture surfaces from interlaminar shear specimens of CFF/PEKK composites: (**a**) RTD; (**b**) ETW.

The failure modes of the in-plane shear specimens of CFF/PEKK composites are illustrated in Figure 8. As shown, upon specimen failure, the fracture surfaces exhibit a 45° or V-shaped morphology, indicative of fiber–matrix cooperative failure. This behavior is attributed to the ±45° layup of the composite specimens, where the fiber orientation aligns with the principal tensile stress direction. Under shear loading, initial matrix cracking occurs, followed by rapid propagation of shear cracks at 45°, ultimately leading to specimen failure [40]. Concurrently, observations at the failure sites reveal fiber breakage (Figure 8b) and delamination (Figure 8c). SEM was employed to examine the microstructure of the fracture surfaces, as depicted in Figure 9. It was observed that the failure modes of the in-plane shear specimens under CTD and RTD conditions were similar, characterized by relatively neat fracture surfaces. In contrast, under ETW conditions, significant fiber pull-out (Figure 9b) was evident on the fracture surfaces, with no apparent delamination or microcracks. These findings suggest that hygrothermal aging deteriorates the interfacial bonding between the resin and fibers, resulting in the debonding of resin matrix and subsequent fiber pull-out. This degradation in interfacial performance, induced by moisture ingress, contributes to the overall material property deterioration.

### 3.4. Interlaminar Shear Properties

**Figure 10:**
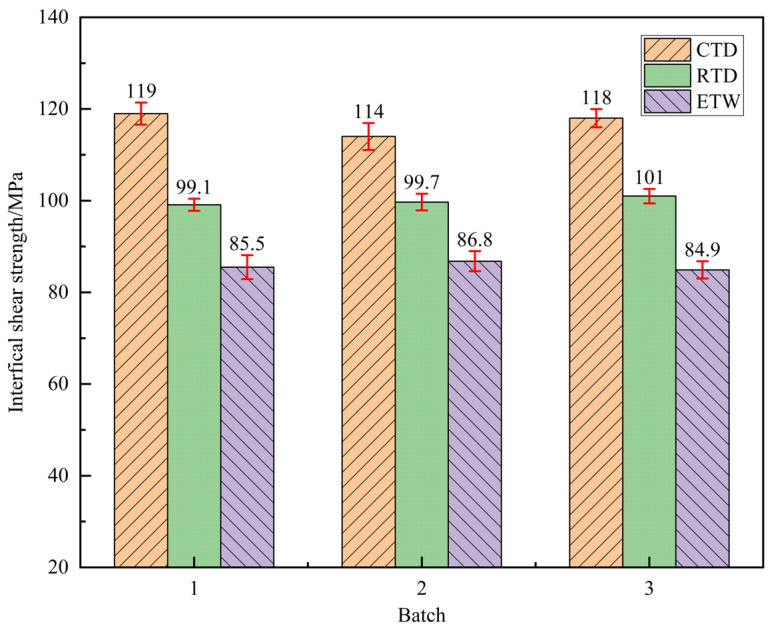
Interfacial shear strength of CFF/PEKK composite under different environmental conditions.

**Figure 11:**
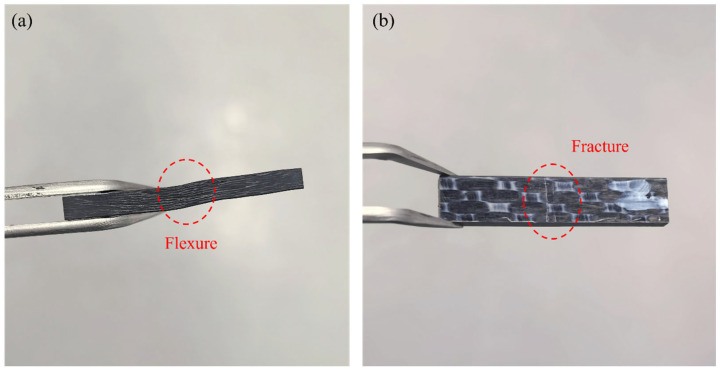
Failed interfacial shear specimens of CFF/PEKK composite: (**a**) flexure; (**b**) fracture.

Interlaminar shear strength testing is a critical parameter for evaluating the interfacial bonding strength of composite materials, directly influencing their interlaminar fracture behavior and durability. The interlaminar shear properties of CFF/PEKK composites under CTD, RTD, and ETW conditions were tested, with results presented in Figure 10. Table 6 shows the retention rates of interlaminar shear strength under different environmental conditions. As indicated by the data, the interlaminar shear strength in the RTD state is significantly higher than that in the CTD state, reaching approximately 117.08% of the CTD value. This enhancement can be attributed to the restricted movement of molecular chains at low temperatures, which increases internal friction and thereby enhances matrix stiffness and strength. Additionally, the difference in thermal expansion coefficients between fibers and matrices may lead to increased thermal stress, strengthening interlaminar bonding and thus improving shear strength. In contrast, under ETW conditions, the interlaminar shear strength decreases significantly, with a retention rate of only 85.8% relative to the RTD state. This decline is primarily due to the pronounced viscoelastic behavior of the matrix in hygrothermal environments, leading to more frequent irreversible sliding under interlaminar shear loading. Furthermore, matrix softening at elevated temperatures reduces overall composite stiffness and impairs shear stress transfer, resulting in a notable decrease in interlaminar shear strength. Observations reveal that the failure modes of interlaminar shear tests under different temperature conditions are relatively similar, with no significant differences noted. Specimen failures predominantly manifest as bending failures (Figure 11), an acceptable failure mode. Notably, some specimens did not exhibit obvious damage after loading, possibly due to extensive interlaminar damage within the composite material, leading to material failure [41]. Comparisons indicate that the interlaminar shear performance of CFF/PEKK composites is more significantly influenced by environmental factors compared to bending and in-plane shear performance, potentially becoming a key limiting factor for its engineering applications. The environmental sensitivity of interlaminar shear properties primarily originates from their strong dependence on the resin matrix and interface. These properties are predominantly governed by the strength of the matrix and interface, whereas other mechanical properties (e.g., tensile, compressive, and flexural) rely more significantly on the fiber strength and the continuity of fiber orientation. The mechanical properties of CFRPs during hygrothermal aging mainly depends on the durability of carbon fiber–resin interface, because the interface is usually the weakest area in the composite material and is more easily attacked and degraded by absorbed moisture [42,43].

## 4. Conclusions

This paper systematically investigates the mechanical properties of CFF/PEKK composite laminates under three conditions: CTD, RTD, and ETW. Additionally, their moisture absorption characteristics, failure modes, and damage mechanisms were also analyzed. The resulting mechanical property data and theoretical insights provide valuable guidance for the practical engineering application of CFF/PEKK composites. Key findings are given as follows. The equilibrium moisture absorption rate of CFF/PEKK composites is approximately 0.27 wt%. In the early stages of moisture absorption, the behavior aligns well with Fickian diffusion, but deviates in later stages. Hygrothermal aging resulted in noticeable fiber pull-out in mechanical specimens, indicating damage to the interfacial performance of the composites. Furthermore, no cracks or delamination were observed. Under CTD conditions, the bending strength of CFF/PEKK composites is approximately 107% of that under RTD conditions, while under ETW conditions, it decreases to about 90% of the RTD value. The bending modulus shows minimal variation across different environmental conditions, with changes within ±3%. the in-plane shear strength of CFF/PEKK composites in CTD conditions increases to approximately 111.52% of the RTD value, while under ETW conditions, it decreases to about 90.06% of the RTD value. The variation in in-plane shear modulus is slightly smaller than that of in-plane shear strength. Specifically, under CTD conditions, the in-plane shear strength is 106.89% of the RTD value, and under ETW conditions, it is 94.39% of the RTD value. Compared to bending and in-plane shear properties, the interlaminar shear strength is more significantly affected by environmental factors. Under CTD conditions, the interlaminar shear strength increases to approximately 117.08% of the RTD value, while under ETW conditions, it decreases to about 85.8% of the RTD value. The primary goal of engineering design is to ensure an optimal balance among the functionality, safety, and cost-effectiveness of materials under extreme conditions. Upon clearly defining the temperature environment and performance requirements, during the material selection phase, designers can select suitable materials by considering the performance characteristics of various composite materials under diverse environmental conditions, as well as their specific application scenarios. The mechanical performance data derived from this study explicitly reveal the temperature-dependent behavior of CFF/PEEK composite materials, thereby offering a solid foundation for material selection across various operating conditions. Furthermore, through the integration of high- and low-temperature data with advanced design methodologies, the reliability and service life of composite material structural components in extreme environments can be substantially improved.

## Figures and Tables

**Table 1:** Lay-up design of CFF/PEKK composite specimens.

Type	Lay-Up Design	Length/mm	Width/mm	Thickness/mm
Weft bending specimen	[0_f_]_8_	150	13	2.4
Warp bending specimen	[90_f_]_8_	150	13	2.4
Interfacial shear specimen	[0_f_]_8_	30	6	3
In-plane shear test	[±45]_2s_	250	25	2.4
Moisture absorption specimen	[0_f_]_8_	50	50	2.4

Notes: Subscript f—fabric prepreg; subscript s—symmetrical layup; layup 0 or 90—angle between the fabric warp direction and the x-axis direction.

**Table 2:** Summary of water absorption parameters.

Material	*n*	*k*/h^−*n*^	*M_m_*/wt%	*D/*(10^−3^ mm^2^/h^−1^)
CFF/PEKK composite	0.48	0.063	0.27	2.01

Notes: *n*—parameter describing the swelling mechanism; *k*—diffusion constant; *h*—specimen thickness, *M_m_*—maximum moisture uptake at equilibrium state; *D*—diffusion coefficient of the composites.

**Table 3:** Retention rate of bending strength of CFF/PEKK composites (%).

Sate	Fiber Direction	First Batch	Second Batch	Third Batch	Average
CTD	Warp	109.13	107.11	106.90	107.71
	Weft	106.37	107.09	108.31	107.26
ETW	Warp	89.61	90.68	89.75	90.01
	Weft	90.64	89.23	88.04	89.30

**Table 4:** Retention rate of bending modulus of CFF/PEKK composites (%).

Sate	Fiber Direction	First Batch	Second Batch	Third Batch	Average
CTD	Warp	102.75	101.56	103.83	102.71
	Weft	101.57	103.21	103.41	102.38
ETW	Warp	97.68	96.89	98.40	97.66
	Weft	97.58	97.52	96.89	97.33

**Table 5:** Retention rate of in-plane shear properties of CFF/PEKK composites (%).

Property	State	First Batch	Second Batch	Third Batch	Average
Strength	RTD	112.67	110.39	111.49	111.52
	ETW	88.67	89.61	91.89	90.06
Modulus	RTD	106.36	107.34	106.97	106.89
	ETW	94.22	95.23	93.71	94.39

**Table 6:** Retention rate of the interfacial shear strength of CFF/PEKK composites.

State	First Batch	Second Batch	Third Batch	Average
RTD	120.08	114.34	116.83	117.08
ETW	86.28	87.06	84.06	85.8

## Data Availability

All supporting data are contained within the manuscript.
